# Cardiac MR fingerprinting with a short acquisition window in consecutive patients referred for clinical CMR and healthy volunteers

**DOI:** 10.1038/s41598-022-23573-3

**Published:** 2022-11-04

**Authors:** Simone Rumac, Anna Giulia Pavon, Jesse I. Hamilton, David Rodrigues, Nicole Seiberlich, Juerg Schwitter, Ruud B. van Heeswijk

**Affiliations:** 1 Department of Radiology, Lausanne University Hospital (CHUV) and University of Lausanne (UNIL), Rue de Bugnon 46, BH08.084, 1011 Lausanne, Switzerland; 2 Division of Cardiology, Cardiovascular Department, Cardiac MR Center, Lausanne University Hospital (CHUV) and University of Lausanne (UNIL), Lausanne, Switzerland; 3 Department of Radiology, University of Michigan, Ann Arbor, MI USA; 4 Faculty of Biology and Medicine, University of Lausanne (UNIL), Lausanne, Switzerland

**Keywords:** Biomedical engineering, Magnetic resonance imaging, Cardiovascular diseases

## Abstract

Cardiac Magnetic Resonance Fingerprinting (cMRF) has been demonstrated to enable robust and accurate T_1_ and T_2_ mapping for the detection of myocardial fibrosis and edema. However, the relatively long acquisition window (250 ms) used in previous cMRF studies might leave it vulnerable to motion artifacts in patients with high heart rates. The goal of this study was therefore to compare cMRF with a short acquisition window (154 ms) and low-rank reconstruction to routine cardiac T_1_ and T_2_ mapping at 1.5 T. Phantom studies showed that the proposed cMRF had a high T_1_ and T_2_ accuracy over a wider range than routine mapping techniques. In 9 healthy volunteers, the proposed cMRF showed small but significant myocardial T_1_ and T_2_ differences compared to routine mapping (ΔT_1_ = 1.5%, P = 0.031 and ΔT_2_ = − 7.1%, P < 0.001). In 61 consecutive patients referred for CMR, the native T_1_ values were slightly lower (ΔT_1_ = 1.6%; P = 0.02), while T_2_ values did not show statistical difference (ΔT_2_ = 4.3%; P = 0.11). However, the difference was higher in post-contrast myocardial T_1_ values (ΔT_1_ = 12.3%; P < 0.001), which was reflected in the extracellular volume (ΔECV = 2.4%; P < 0.001). Across all subjects, the proposed cMRF had a lower precision when compared to routine techniques, although its higher spatial resolution enabled the visualization of smaller details.

## Introduction

Over the past decade, the field of cardiac magnetic resonance (CMR) saw an increasing interest in the measurement of myocardial T_1_ and T_2_ relaxation times^1–3^, and multiple studies have now shown the added diagnostic value of myocardial T_1_ and T_2_ values^5^. Native T_1_ mapping (i.e. without a gadolinium-based contrast agent—GBCA) has become a widely used technique to detect interstitial fibrosis^6–8^, while T_1_ mapping after the administration of GBCA provides unique additional prognostic information through the estimation of the extracellular volume (ECV) fraction^9–11^. Increased myocardial T_2_ values similarly provide a quantitative measure of myocardial edema in various pathologies^12,13^.

While these findings are highly encouraging, the clinical adoption of parametric mapping is hindered by relatively long acquisition times and ongoing discussions about accuracy, precision, robustness, and sensitivity^5^. These challenges have encouraged the exploration of new mapping techniques^14–19^. It would especially be desirable to develop a fast and reliable method capable of simultaneously quantifying both T_1_ and T_2_ relaxation times, in order to save time and to remove the bias that one parameter can have on the estimation of the other.

Magnetic Resonance Fingerprinting (MRF) has been proposed^20^ as an alternative approach to parameter mapping that enables the simultaneous quantification of multiple relaxation times. The idea behind MRF is to deliberately vary the pulse sequence parameters in order to induce a unique signal evolution that depends exclusively on the tissue properties under investigation (e.g. T_1_ and T_2_). After acquiring data with a highly undersampled k-space sampling trajectory, a pattern matching algorithm is used to match each voxel’s time course to an entry in a dictionary containing simulated MRF time courses. The best-matching dictionary entries are used to populate the parametric maps with the corresponding values. The accuracy and efficiency of this approach have been demonstrated for stationary tissues such as the brain^20,21^ and prostate^22^.

The use of MRF for parametric mapping in the heart, however, faces additional challenges due to respiratory and especially cardiac motion. In order to address these challenges, cardiac MRF (cMRF) makes use of breath holds and electrocardiographic (ECG) triggering. Similar to established myocardial mapping techniques such as MOLLI^23^, SASHA^16^ or T_2_-prepared bSSFP^24^, cMRF data are acquired during an acquisition window in the mid-diastolic phase of each heartbeat, over the span of a single breath hold. This results in a shorter and interrupted acquisition, which limits the amount of signal that can be matched to the dictionary as well as the variation in the signal, which is required for accurate pattern matching. At the same time, since the duration of the mid-diastolic quiescent period depends on the subject’s heart rate, a longer acquisition window is not always possible in the presence of pathologies. Most MRF applications in stationary organs (e.g. brain) use sequence lengths of 1000 TRs or more, and many prior cMRF studies have used a scan duration of 15–16 heartbeats with a 250 ms acquisition window, leading to approximately 750 TRs that can be used for fitting. Reducing the acquisition window to 150 ms would be especially desirable in patients with high heart rates. However, this would result in a sequence length of approximately 400 TRs, which limits the amount of cMRF data that can be collected and in turn may result in a loss of measurement precision. To counter the expected loss in precision caused by shortening the acquisition window, the intrinsic redundancy and similarity of the magnetization time courses can be exploited. Instead of using the full dictionary, the redundancy allows for the use of a low-rank approximation of the dictionary, which is a highly efficient regularization constraint for iterative reconstruction. Such a low-rank iterative reconstruction can thus be used to reduce noise and aliasing artifacts and to compensate for the precision loss^25–27^.

The feasibility of a cMRF sequence that combines such a short acquisition window and low-rank reconstruction has recently been presented in healthy volunteers^28^. Therefore, the goal of this study was to assess the robustness of the technique in a routine clinical setting by comparing its accuracy and precision to routine myocardial T_1_ and T_2_ mapping in a cohort of patients referred for clinical CMR for a wide range of indications.

## Results

### Phantom studies

**Figure 1:**
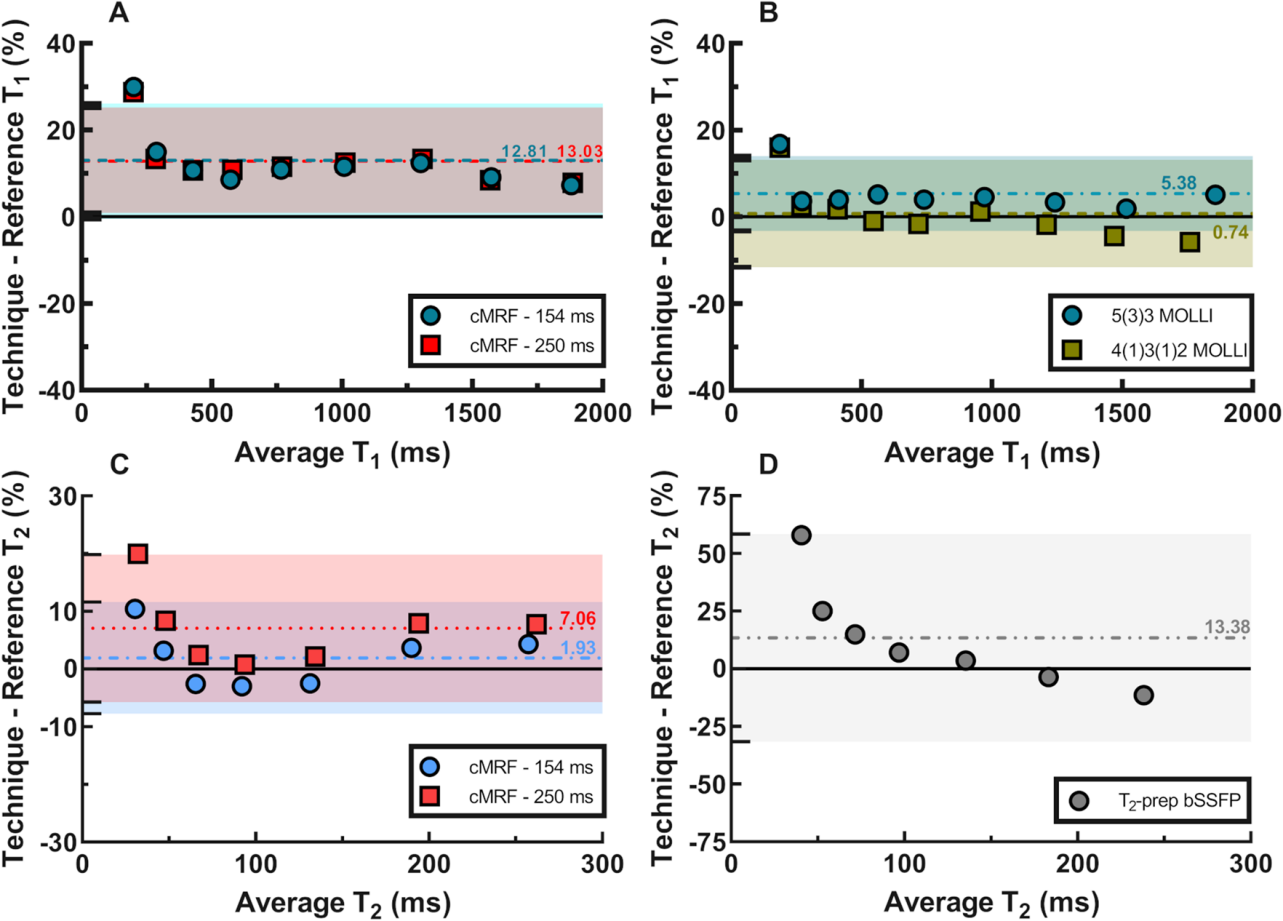
Accuracy of cMRF compared to reference techniques in the ISMRM-NIST phantom. (**A**) Both cMRF versions showed good agreement with one another and the bias was around 10% across a wide range of T_1_ values. (**B**) The two MOLLI sequences maintained a low bias, with 5(3)3-MOLLI performing slightly better at higher T_1_ values, as expected. (**C**) The cMRF T_2_ measurement were consistent between the two variations, with the shorter acquisition window showing lower bias overall. (**D**) T_2_-prepared bSSFP had higher error than cMRF, likely due to the high T_1_ values of the NIST phantom. The 95% confidence intervals are reported as matching semi-transparent colored bands in each plot, and the same color scheme was used to report the biases.

When considered over the entire tested range, the phantom mapping demonstrated similar or higher T_1_ and T_2_ accuracy for cMRF than the routine mapping techniques (Fig. 1A,B, Suppl. Fig. 1). In the T_1_ vials, the average bias and 95% limits of agreement obtained from the Bland–Altman analysis were equal to 12.81 ± 13.34% for cMRF with a 154 ms acquisition window and 13.03 ± 12.15% for the cMRF sequence with a 250 ms acquisition window (Fig. 1A). The two MOLLI variants had overall smaller biases and comparable limits of agreement, and the errors were equal to 5.38 ± 8.63% and 0.78 ± 12.39% for 5(3)3-MOLLI and 4(1)3(1)2-MOLLI, respectively (Fig. 1B). The same analysis was performed for T_2_ relaxation times and confirmed the overall good agreement with reference measurements. The biases and limits of agreement were respectively 1.55 ± 9.21% and 6.76 ± 11.93% for the two variants with shorter and longer acquisition window (Fig. 1C). T_2_-prepared bSSFP (Fig. 1D) showed a similar bias, but a considerably larger confidence interval: 8.76 ± 48.9%. The short-acquisition-window cMRF reconstruction time was 297 ± 20 s.

For both the T_1_ and T_2_ relaxation times, the heart-rate influence phantom study showed no significant variation or trend in the resulting T_1_ and T_2_ values as the R-R interval was increased (Suppl. Fig. 2). For the T_1_ relaxation times, the CoV for both cMRF variants was below 2%, which was comparable to that of 5(3)3-MOLLI and slightly better than that of 4(1)3(1)2-MOLLI. For the T_2_ relaxation times, the CoV was higher, and ranged between 2 and 8% across the relevant T_2_ values (Suppl. Fig. 2C,D). For both relaxation times, no significant difference was observed between the cMRF sequences with the shorter and longer acquisition windows. Moreover, for both cMRF variations, and both T_1_ and T_2_, the ANCOVA analysis found no significant difference between the linear regression slopes obtained at different heart-rates (all P values > 0.05, Suppl. Fig. 3A–D).

### Healthy volunteers

**Figure 2:**
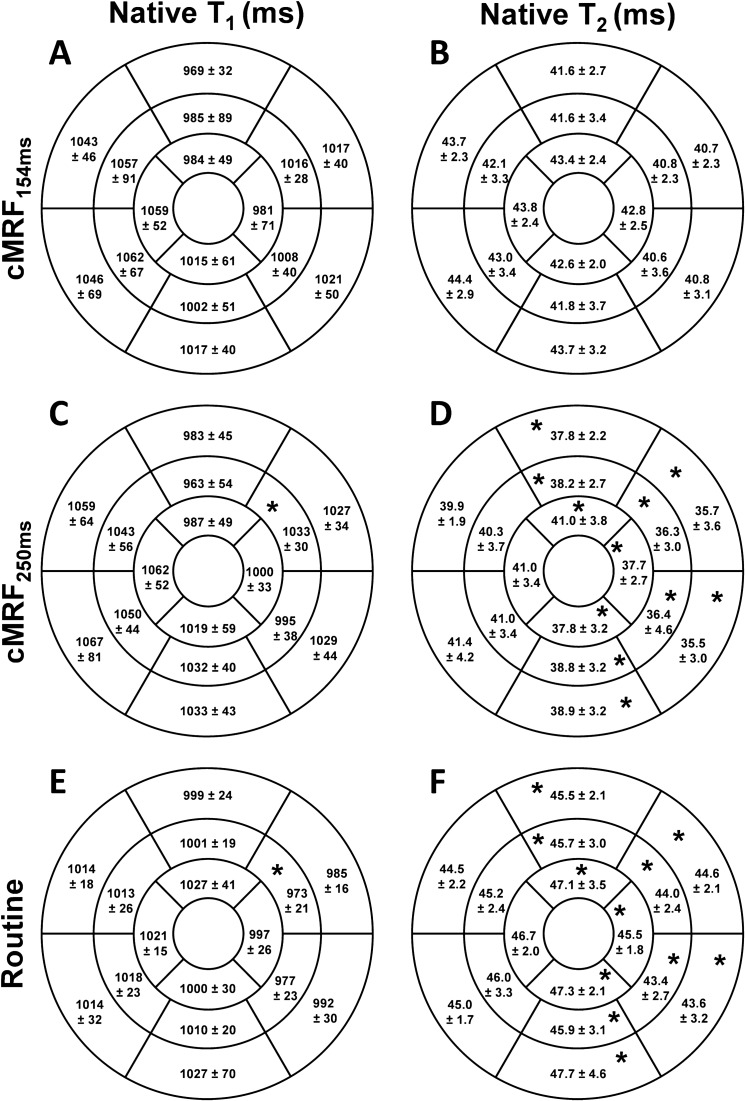
Circumferential polar plots of the myocardial relaxation times in 9 healthy volunteers. Myocardial areas were segmented according to the AHA guidelines^29^. Each segment reports the average relaxation time and its standard deviation across all volunteers in milliseconds. cMRF with a shorter acquisition window is shown in the first row (**A** and **B**); cMRF with a longer acquisition window is shown in the second row (**B** and **C**) routine T_1_ and T_2_ values are reported in the third row (**E** and **F,** respectively). No significant differences were found between the two variations of cMRF as well as between cMRF with a short acquisition window and routine techniques. Significant differences were found exclusively in the mid and apical slices between routine and cMRF with a longer acquisition window, as highlighted (*P < 0.05).

In 9 healthy volunteers (age 25 ± 2 years, 66% female, heart rate 69 ± 17 bpm, ranging from 53 to 111), cMRF resulted in similar relaxation times with a slightly lower precision compared to routine techniques (Fig. 2). The inter-observer variability showed good agreement between two different observers: whole-heart T_1_ and T_2_ values were not significantly different for cMRF_150ms_ (P = 0.148 and P = 0.535, respectively). A small but significant bias was found when analyzing the cMRF_250ms_ T_1_ maps (P = 0.008, bias = 8.6 ms), while whole-heart T_2_ distributions did not differ significantly between the two observers (P = 0.177).

Averaged over the three slices and the nine volunteers, the myocardial T_1_ values were slightly higher and varied more for both versions of cMRF when compared to MOLLI (cMRF_154ms_: 1020 ± 45 ms; cMRF_250ms_: 1026 ± 34 ms; MOLLI: 1005 ± 20 ms). Both techniques also differed significantly from MOLLI (P = 0.031 and P = 0.002, respectively for cMRF_154ms_ and cMRF_250ms_). No significant difference was found between the two cMRF variants (P > 0.999). When compared on a per-segment basis, the T_1_ difference was significant in the septum of the mid slice between cMRF_250ms_ and MOLLI (segment 12; Fig. 2C–E). No significant difference was observed between the two cMRF variants and between cMRF_154ms_ and MOLLI. Overall T_2_ values, on the contrary, were lower in cMRF compared to T_2_prep-bSSFP, and confirmed the trend observed in vitro (cMRF_154ms_: 42.4 ± 2.4 ms; cMRF_250ms_: 38.5 ± 2.6 ms; T_2_prep-bSSFP: 45.4 ± 2.0 ms). All whole-heart distributions were significantly different (P < 0.001 for all comparisons). When comparing the segments, significant difference was found between cMRF_250ms_ and T_2_prep-bSSFP in the lateral part of all three slices (Fig. 2C–F). The per-segment precision was higher in routine techniques: cMRF with 154 ms acquisition window resulted in a T_1_ CoV of 7.9 ± 2.0% and T_2_ CoV of 7.7 ± 3.3%. cMRF with 250 ms acquisition window resulted in a T_1_ CoV of 6.8 ± 1.8% and T_2_ CoV of 8.1 ± 3.2%. MOLLI resulted in a T_1_ CoV of 5.3 ± 0.9% and T_2_prep-bSSFP in a T_2_ CoV of 6.5 ± 2.0%.

### Patient scans

Scans were completed in n = 61 patients referred for CMR (Suppl. Fig. 4); in one patient, all mapping failed due to the presence of an implantable cardioverter-defibrillator (ICD). Native cMRF was completed in 59 patients; in one case, it failed due a sudden deep breath of the patient (not noticed until the maps were reconstructed). Native routine T_1_ and T_2_ mapping were completed in 58 and 13 subjects and failed in two and one cases respectively due to bSSFP banding artifacts. Post-contrast cMRF and routine T_1_ mapping were completed in 39 subjects, while for the latter, two acquisitions failed due to bSSFP banding artifacts. This resulted in n = 59 for native cMRF, n = 58 for native T_1_ mapping, n = 13 for native T_2_ mapping, n = 39 for post-contrast cMRF, and n = 37 for post-contrast T_1_ mapping.

**Figure 3:**
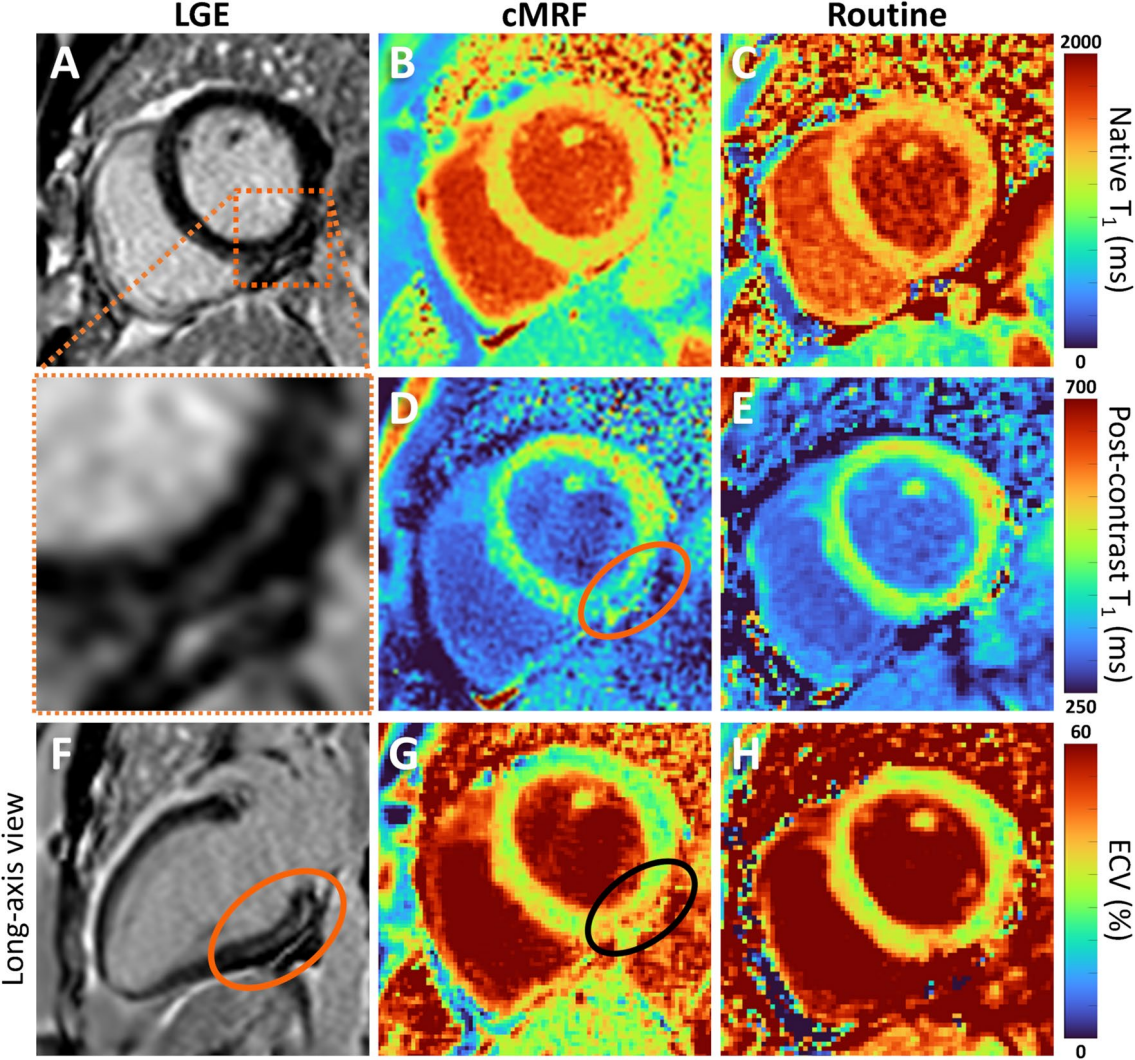
T_1_ and T_2_ maps in a 40 y.o. patient with viral myocarditis and old sub-epicardial scar in the basal infero-lateral and inferior wall. (**A**,**F**) Short- and long-axis late-gadolinium-enhanced (LGE) images showing the presence of the subtle subepicardial scar. (**B**,**C**) Native T_1_ maps. The routine T_1_ map had slightly higher overall T_1_ myocardial values. (**D**,**E**) T_1_ maps 20–25 min after contrast agent injection. We can observe that the small non-transmural subepicardial lesion is easier to identify in the cMRF scan. (**G**,**H**) ECV maps obtained with cMRF and routine technique. The sub-epicardial scar is visible in the cMRF ECV map.

**Figure 4:**
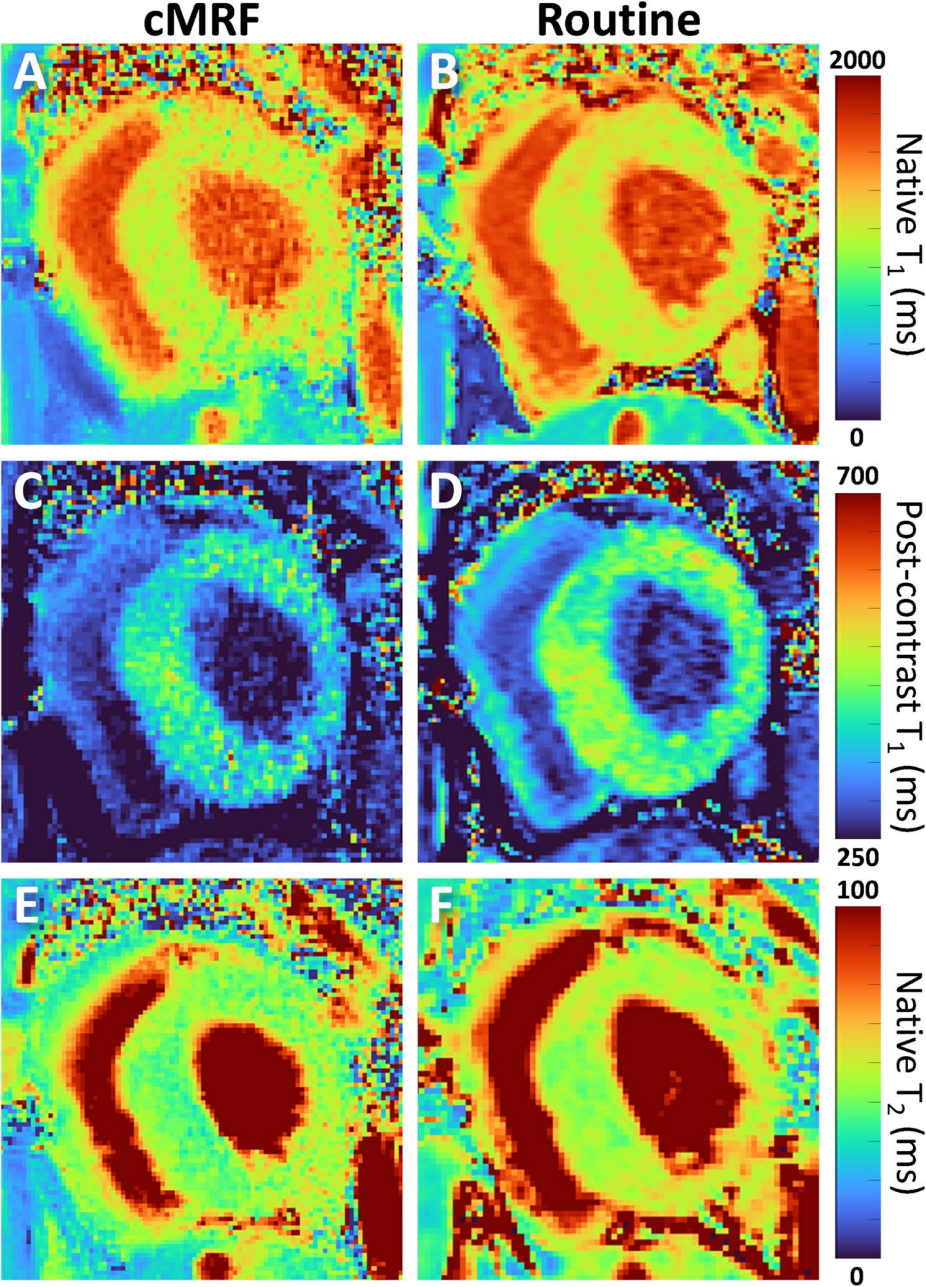
Respiratory drift resulted in noisier cMRF T_1_ and T_2_ maps in a 35 y.o. patient with hypertrophic CMP. The patient did not comply with the breath holding protocol and slowly breathed out during several acquisitions. (**A**,**B**) T_1_ maps before contrast agent injection. The patient complied with the very first breath hold (the routine T_1_ map), but not the subsequent cMRF: its T_1_ map appears noisy and its borders are poorly defined. The myocardial T_1_ values are 1079 ± 99 ms for cMRF and 1119 ± 69 ms for MOLLI. (**C**,**D**) T_1_ maps 20–25 min after contrast agent injection. Despite some respiratory drift, the routine T_1_ map source images were correctly aligned during registration and resulted in a precise map. Also note the higher T_1_ values in the myocardium of the routine map (404 ± 39 ms for cMRF and 447 ± 29 ms for MOLLI). (**E**,**F**) Native T_2_ maps. Slightly higher regional variability can be observed in the routine map, and the total myocardial area appears smaller due to poor registration of the source images. The averages across the myocardium were 46 ± 5 ms for cMRF and 51 ± 5 ms for T_2_prep-bSSFP.

Overall, we observed a robust performance of cMRF with a low number of failed acquisitions. We anecdotally observed that the higher spatial resolution of cMRF allowed for the easier identification of small and non-transmural lesions in several patients (Fig. 3, Suppl. Fig. 5). cMRF also still resulted in accurate maps in the presence of large respiratory drifts, albeit producing visibly noisier maps (Fig. 4).

**Figure 5:**
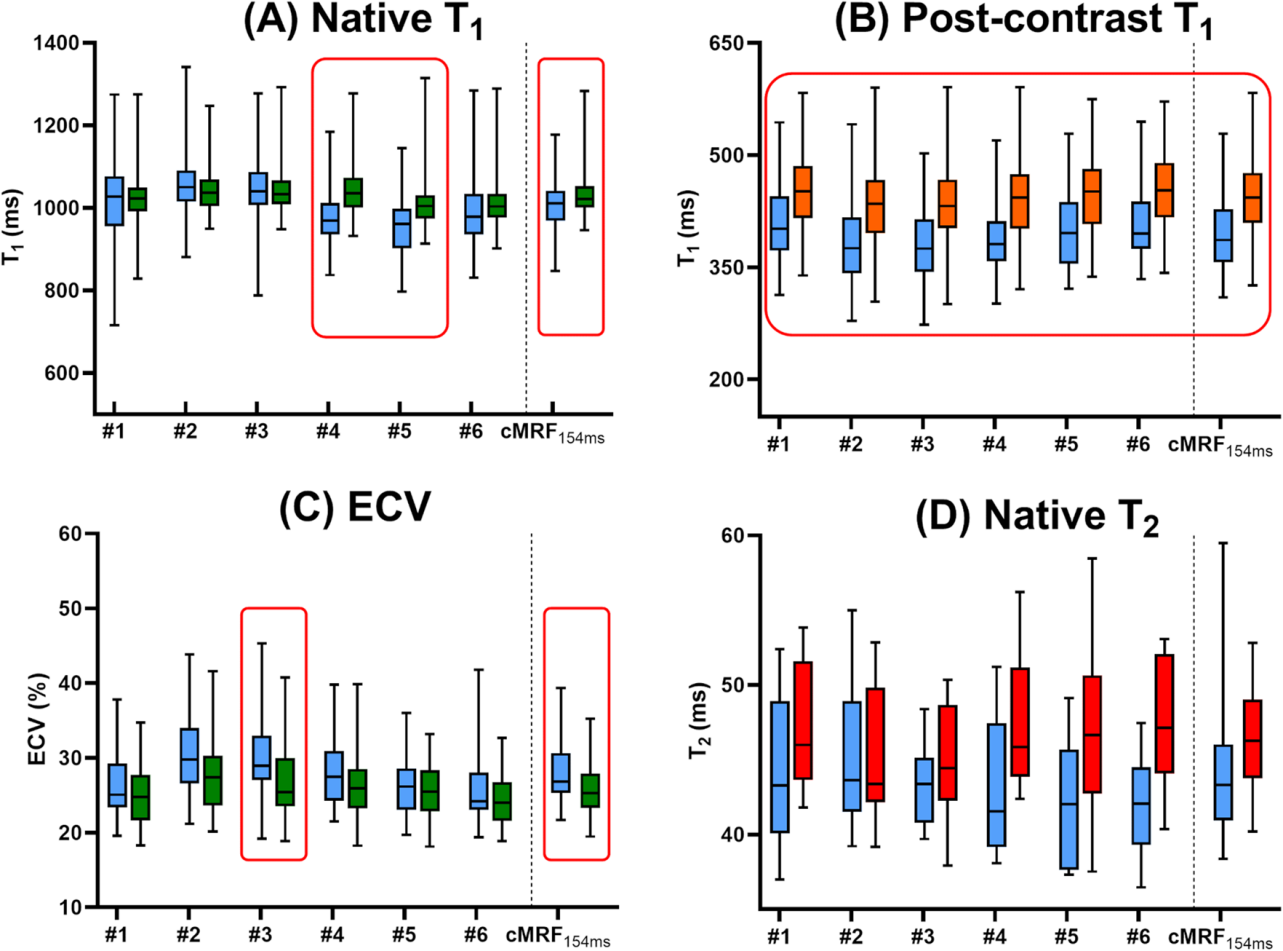
Box and whiskers plots of segmental relaxation times in the myocardium of the patient group. Boxes are medians with interquartile ranges; whiskers indicate the minimum and maximum values. The segments that showed a statistically significant difference (P < 0.05) are outlined in red. (**A**–**C**) Native T_1_, post-contrast T_1_ and synthetic ECV measured with short-acquisition-window cMRF (left, blue) and MOLLI (right segment, orange and green). cMRF and routine techniques have similar ranges. (**D**) Native T_2_ measured with cMRF (left, blue) and T_2_prep bSSFP (right, red). The numbers indicate the AHA segment number and ‘avg’ is the average across the entire myocardium.

**Figure 6:**
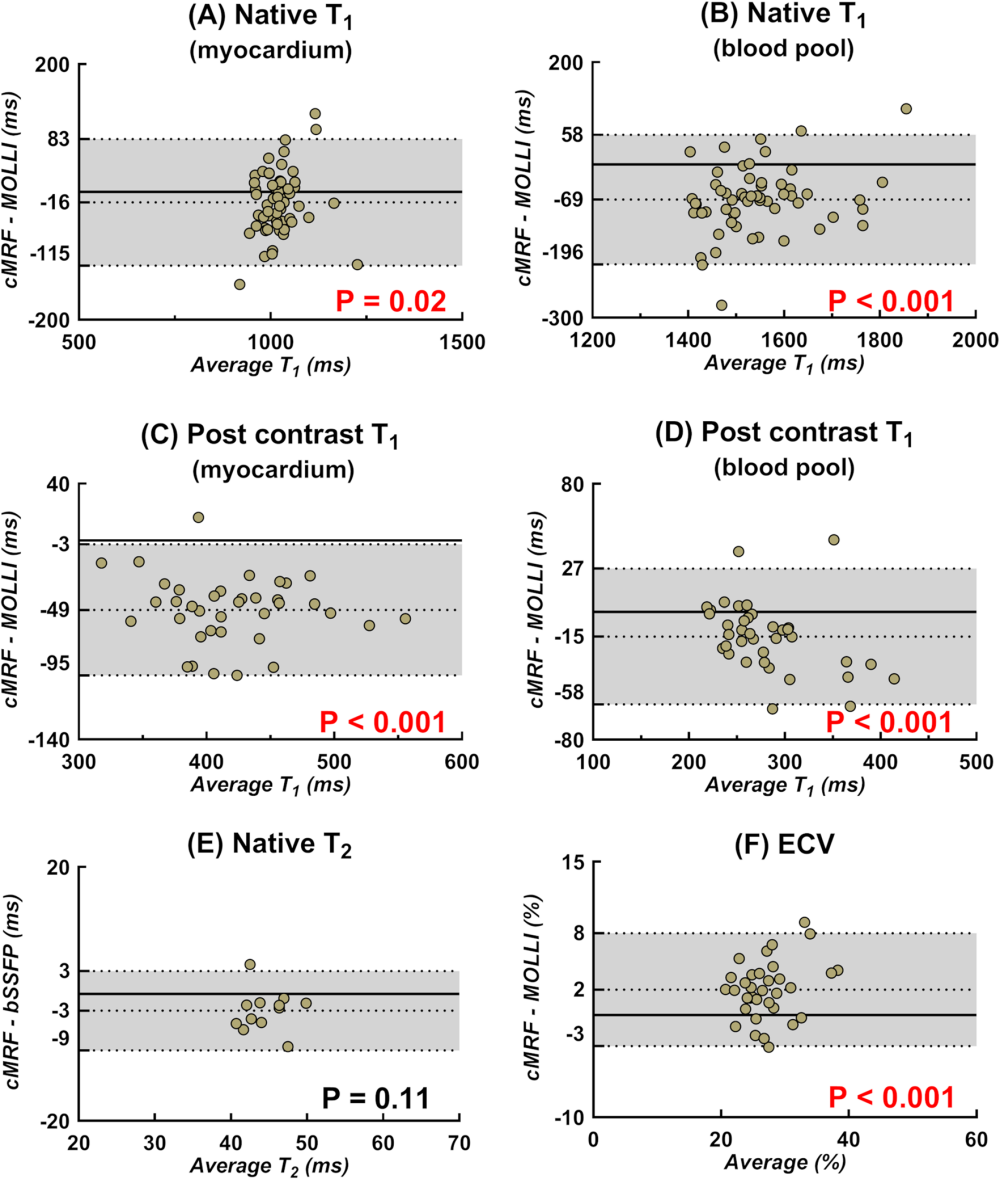
Bland–Altman analyses of short-acquisition-window cMRF and routine techniques for the entire visible left-ventricular myocardium and the left-ventricular blood pool in patients. (**A**,**B**) Native T_1_ relaxation times obtained with cMRF vs. 5(3)3-MOLLI in the myocardium and blood pool areas, respectively. (**C**,**D**) Post-contrast T_1_ relaxation times obtained with cMRF vs. 4(1)3(1)2-MOLLI. A significant bias was observed, while the difference in the post-contrast LV blood T_1_ was smaller between the two techniques. (**E**) Native T_2_ relaxation times obtained with cMRF vs. T_2_-prepared bSSFP. (**F**) Synthetic ECV calculated with cMRF vs. MOLLI. P-values indicate the significance of the unpaired t-test; P < 0.05 is highlighted in red. None of the measured parameters showed significant trends or changes with increasing value.

**Figure 7:**
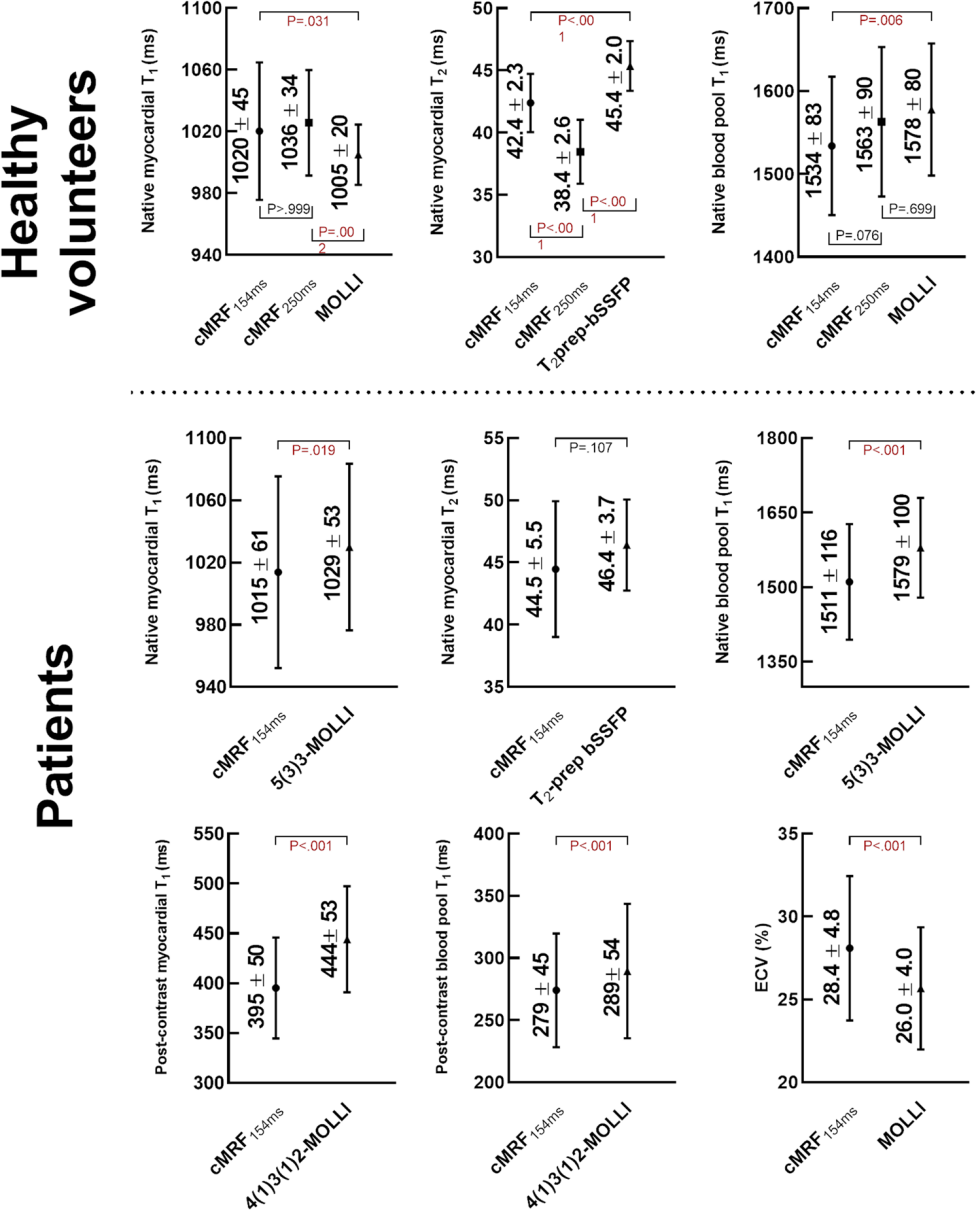
Myocardial relaxation times and ECV measured with short-acquisition-window cMRF and routine techniques. Similar native relaxation times were measured with all techniques in both the healthy volunteers and patients. However, the bias in the post-contrast myocardial T_1_ values was higher between cMRF and MOLLI, which together with similar post-contrast blood T_1_ values resulted in slightly different ECV estimations. All P values are reported on the plots.

The agreement between cMRF and routine techniques seen in the phantom and healthy volunteer studies was confirmed in the native myocardial T_1_ relaxation times (cMRF: 1015 ± 61 ms; MOLLI: 1029 ± 53 ms; ΔT_1_ = 1.6%; P = 0.02). A small but significant difference was found in the inferior and inferolateral segments as well as in the blood pool, where cMRF measured lower T_1_ values than MOLLI (Figs. 5A, 6A,B, 7). cMRF showed a substantial difference from the routine post-contrast myocardial T_1_ value (cMRF: 395 ± 50 ms; MOLLI: 444 ± 53 ms; ΔT_1_ = 12.3%; P < 0.001, Fig. 6C), which was consistent in all segments (Fig. 5B). Contrary to this, the post-contrast blood-pool T_1_ value had similar average values (cMRF: 279 ± 45 ms; MOLLI: 289 ± 54 ms; ΔT_1_ = 3.6%; P < 0.001, Fig. 6D). The different post-contrast myocardial (and the native blood pool) T_1_ values were reflected in a higher ECV than that established with MOLLI (cMRF: 28.4 ± 4.8%; MOLLI: 26.0 ± 4.0%; ΔECV = 8.4%; P < 0.001, Figs. 5C, 6F). In the myocardium, T_2_ values obtained with cMRF were marginally lower compared to those obtained with T_2_-prep bSSFP, but no significant difference was observed (cMRF: 44.5 ± 5.5 ms; T_2_-prep bSSFP: 46.4 ± 3.7 ms; ΔT_2_ = 6.1%; P = 0.11; Figs. 5D, 6E). The segmental precision of cMRF was again lower than that of the routine techniques for the native T_1_ (cMRF: 12.0 ± 3.7%; MOLLI: 6.8 ± 1.5%), post-contrast T_1_ (cMRF: 10.6 ± 3.1%; MOLLI: 7.4 ± 2.5%), and T_2_ values (cMRF: 12.3 ± 2.9%; T_2_prep-bSSFP: 9.1 ± 1.7%); all with P < 0.001. Finally, none of measured relaxation times (routine or cMRF) resulted in a significant linear regression with the individual patient heartrate (P > 0.14 for all, Suppl. Fig. 6).

## Discussion

Cardiac MR fingerprinting with a short acquisition window and low-rank reconstruction^28^ was characterized in vitro, in healthy volunteers and as part of a clinical CMR protocol to assess its accuracy and precision compared to routine T_1_ and T_2_ mapping techniques.

In vitro, cMRF had slightly lower accuracy than routine techniques when tested against reference relaxation times spanning from very low (90 ms) to very high (1900 ms) T_1_ values. T_1_ values in the native myocardial range were higher for both cMRF sequences, while excellent agreement was observed for the entire T_2_ range. Despite the small size of these differences, they were interestingly opposite to what was found in vivo, where good agreement was found between the native T_1_ values (around 1000 ms) of both healthy volunteers and patients, and lower values compared to MOLLI were found after GBCA injection. We observed high consistency between the longer and shorter cMRF sequences both in vitro and in vivo, with the measured relaxation times often overlapping. The study of heart-rate variability influence in the phantom also showed consistent values for both techniques across a wide range of relaxation times and simulated heart-rates. The slightly higher heartrate CoVs for the cMRF techniques than the routine technique over the entire heartrate range are still lower than a single segmental myocardial T_2_ standard deviation observed in vivo, from which we estimate that they can be ignored.

The differences between the cMRF and the IR-TSE reference likely have several causes, such as using a TSE instead of an SE sequence, and not including the slice profile of the RF pulse^30^, B_0_ and B_1_^+^ inhomogeneities^31^, and inversion efficiency^17^ in the fitting model of IR-TSE reference, which might lower its accuracy. It should be noted that the RF pulse profile is included in the cMRF dictionary, and that the presented cMRF technique used adiabatic inversion and refocusing pulses, as opposed to the IR-TSE and SE techniques, making it less susceptible to B_1_ inhomogeneities. Moreover, the T_1_/T_2_ ratios in the phantom vary over a much broader range than in vivo. Given these sources for mismatching and discrepancies, it is encouraging to note that the overestimation in vitro still consistently remained below 10%.

Both in the healthy volunteer group and in the patients, cMRF showed a high agreement and comparable inter-patient standard deviations when compared to MOLLI and T_2_-prep bSSFP. In the patient group, the majority of the subjects was referred with suspected and not confirmed disease, and did not have significantly altered myocardial relaxation times with any of the used techniques. The global average across all patients was therefore close to that of the healthy volunteers, with virtually identical average T_1_ and T_2_ values measured in both groups. When a subgroup of patients with a specific disease was selected (not reported here), this also held true, although small sub-endocardial infarctions were more clearly delineated in the cMRF maps, most likely due to the higher spatial resolution.

The relaxation times observed here are in line with previous studies where the accuracy and precision of cMRF were established^32–34^. In particular, Hamilton et al. found moderately lower relaxation times and equivalent standard deviations (964 ± 71 ms and 41.2 ± 4.2 ms for T_1_ and T_2_, respectively) in fifty healthy volunteers at 1.5 T^32^. In twenty-four patients with suspected inflammatory cardiomyopathy^33^, cMRF resulted in very similar myocardial T_1_ = 1028 ± 64 ms and higher T_2_ = 52.8 ± 3.8 ms. The same comparison between cMRF and routine techniques was performed pre- and post-contrast in six hypertrophic cardiomyopathy patients and 12 healthy subjects^34^: small differences in myocardial relaxation times were found between routine techniques and cMRF, although both methods easily allowed for the distinction between patients and healthy subjects. Interestingly, larger differences were found in the ECV estimation, where cMRF measured consistently higher percentages in both groups.

The larger difference in the post-contrast myocardial T_1_ values is the main cause of difference in ECV estimation, although it should be noted that Treibel et al. obtained the empirical formula to calculate the hematocrit specifically from MOLLI sequences^35^. The timings of each post-contrast acquisition with respect to the GBCA injection were verified, as was the accuracy in the delineation of the myocardial area: both were not found to be significantly different between cMRF and routine techniques after verification by a second observer. To the best of our knowledge, neither of these factors alone could substantially influence the myocardial T_1_ estimation, and an adequate explanation for the mismatch remains to be found.

In both subject groups, the inter-subject standard deviations of cMRF and routine techniques were similar and between 5 and 12% of the measured value. The individual segmental precision of cMRF is in line with previous studies^32,36,37^. While it is lower than its routine counterparts, this is balanced with the 1.5-fold to 2.4-fold smaller pixel size of cMRF as well as the smoothing effects of the non-rigid motion registration techniques used for routine mapping techniques^38^. This suggests that it might be of interest to integrate a spatial regularization filter or a denoising step in the reconstruction pipeline of cMRF data^27^, while the cMRF spatial resolution could be lowered to that of the routine scans, if desired. This may result in a higher precision for cMRF, while leaving the other advantages of cMRF intact: acquisition of both maps at half the time, using the same protocol for all T_1_ (and T_2_) ranges, and intrinsic co-registration of the T_1_ and T_2_ maps^5^. If a larger spatial coverage is desired, the proposed shorter acquisition window could for example be combined with a simultaneous multislice (SMS) acquisition^39^, although the accuracy and precision of such a technique would need to be established. Similarly, a 3D free-breathing technique^40^ could be used if extra scan time can be allocated to obtain whole-heart coverage, which may especially be of use in diseases that manifest in unpredictable patchy patterns.

The main limitation of this study lies with the broad variety of scanned patients. While this variety is a strength of the study in that it enabled us to characterize cMRF in a cohort that represents a real heterogeneous clinical population, it did not result in a narrow range of relaxation times that was significantly different from healthy volunteers. This effect was further exacerbated by the patients only having suspected and not confirmed cardiac disease, and by the standardized segmental analysis that might average out any small regional T_1_ or T_2_ elevations. Therefore, it might be of interest to further characterize this technique in cohorts of patients with a specific and confirmed disease. In two of the aforementioned studies^33,34^, a blinded evaluation of cMRF against MOLLI and T_2_prep-bSSFP was performed, where cMRF compared favorably against both techniques. This might also be incorporated in future work to provide a better overall understanding of the diagnostic potential of cMRF.

In conclusion, the comparison of cMRF with a short acquisition window with routine myocardial T_1_ and T_2_ mapping techniques showed a robust performance in vitro, in healthy volunteers, and in a group of CMR patients covering a broad range of pathologies. The T_1_ and T_2_ maps generated from the single cMRF acquisition were generally comparable in accuracy, slightly less precise, and had a higher spatial resolution than those obtained with routine T_1_ and T_2_ mapping techniques. A notable exception was found in a lower estimation of the post-contrast myocardial T_1_ relaxation time, which led to a small but significantly higher ECV.

## Materials and methods

The local ethical committee (Commission cantonale d'éthique de la recherche sur l'être humain du canton de Vaud CER-VD, Lausanne, Switzerland) approved all in vivo studies under protocol number 2018-00656 (principal investigator JS), all studies were performed in accordance with the relevant guidelines and regulations, and all participants provided written informed consent. All scans were performed on a clinical 1.5 T MR scanner (MAGNETOM Sola, Siemens Healthcare, Erlangen, Germany), using an 18-channel chest coil combined with a 12-channel spine coil.

### Phantom study

The accuracy of the proposed cMRF technique was first assessed using the ISMRM-NIST phantom (QalibreMD, Boulder, CO, USA), a 200-mm spherical phantom with two layers comprised of 14 spheres, one with a wide range of T_1_ values and one with a wide range of T_2_ values. The T_1_ array was created by NiCl_2_-doped deionized water, while the T_2_ array spheres are filled with MnCl_2_-doped deionized water. A single slice was planned through the T_2_ layer of spheres (n = 14), since it contains a broad T_1_ and T_2_ range that mimics relevant physiological values. Because all sequences were designed to be accurate within physiological ranges, T_1_ values lower than 90 ms and higher than 1900 ms were discarded in the analysis. For the same reason, we considered T_2_ values ranging from 30 to 240 ms. For completeness, the measurements obtained in all 14 vials are reported as supplementary material.

Parameters for the cMRF pulse sequence included: spiral readout, 15-heartbeat breathhold, 48-fold undersampling, field of view (FOV) = 300 × 300 mm^2^, pixel size = 1.6 × 1.6 mm^2^, slice thickness = 8 mm, 2.5 µs dwell time, and sampling bandwidth ± 200 kHz. The repetition time (TR) was fixed at 5.3 ms, while the flip angle was varied between 4° and 25°. To facilitate the distinction between different relaxation times, the cMRF sequence incorporates four different magnetization preparation modules: an adiabatic inversion recovery and three adiabatic T_2_ preparations^41^ with echo times of 30, 50 and 80 ms. Two variations of the cMRF sequence were considered and compared: one with a longer acquisition window = 250 ms and 48 undersampled image acquisitions per heartbeat and one with the shorter acquisition windows = 154 ms and 29 undersampled image acquisitions per heartbeat. We will differentiate between the two variants by indicating the acquisition window duration in a subscript.

A low-rank reconstruction was used to reduce the dimensionality of the generated dictionaries and reduce aliasing artifacts in the MRF images^26,27,39^. Similar to previous work, this approach compresses the dictionary along the time dimension by calculating the singular value decomposition of the dictionary and retaining the first five singular values. Images in this low-dimensional subspace are reconstructed using nonlinear conjugate gradient descent with both ${l}_{1}$ wavelet regularization and locally low-rank patch regularization with a patch size of 8 × 8. The dictionary took the slice profile and imperfections due to the efficiency of preparation pulses into account^30^. All reference and cMRF maps were reconstructed in MATLAB 2019a (The MathWorks, Natick, MA, USA; http://www.matlab.com) on a desktop PC with an i9-9900 CPU @3.10 GHz and integrated Intel UHD Graphics 630, and the reconstruction time was recorded.

As recommended in the phantom manual, reference T_1_ relaxation times were obtained with an inversion-recovery turbo spin echo (IR-TSE) pulse sequence with nine inversion times (35–3000 ms), TE = 7.9 ms, TR = 4500 ms, and spatial resolution = 1 × 1 mm^2^. A spin-echo (SE) pulse sequence was used to determine reference T_2_ values, with 25 echo times (TEs) ranging from 10 to 400 ms, pixel bandwidth = 279 Hz, TR = 5000 ms, spatial resolution = 1 × 1 mm^2^, and slice thickness = 6 mm.

Three clinical routine parametric mapping techniques were also characterized: 5(3)3-MOLLI (21) for T_1_ mapping in the native T_1_ range, 4(1)3(1)2-MOLLI^4^ for T_1_ mapping in the post-contrast T_1_ range (both variants used heartbeats for their timing), and T_2_-prepared bSSFP for T_2_ mapping^24^. 5(3)3-MOLLI and T_2_-prep bSSFP had FOV = 300 × 300 mm^2^, pixel size = 2.0 × 1.6 mm^2^ interpolated to 1.6 × 1.6 mm^2^, slice thickness = 8 mm. 4(1)3(1)2-MOLLI was performed with FOV = 306 × 360 mm^2^, pixel size = 2.1 × 1.4 mm^2^ interpolated to 1.4 × 1.4 mm^2^, slice thickness = 8 mm. Both MOLLI sequences and T_2_-prep bSSFP had TE = 1.1 ms. Acquisition window durations for cMRF, 5(5)3-MOLLI, 4(1)3(1)2-MOLLI and T_2_-prep bSSFP were 154 ms, 194 ms, 194 ms, and 144 ms, respectively. Routine maps were reconstructed on the scanner.

Multiple synthetic heart-rates were simulated in order to study the influence of heart-rate variability on the cMRF techniques. The R-R intervals varied from 600 to 1300 ms, at 100 ms intervals (8 in total).

### Healthy volunteer study

We compared both cMRF versions to 5(3)3-MOLLI and T_2_-prepared bSSFP in 9 healthy volunteers. Three short-axis views were acquired with each technique (apex, middle, and base of the left ventricle) with breath holds in end-expiration. All three techniques were acquired with the same parameters used for the phantom study. After manual segmentation, the myocardium was subdivided according to the American Heart Association guidelines^29^.

### Patient study

The cMRF with a shorter acquisition window was inserted in a clinical protocol for myocardial viability assessment in 61 consecutive patients referred for CMR at the Lausanne University Hospital (CHUV).

Patients (age 57 ± 14 years, 28% female, heart rate 66 ± 12 bpm ranging from 52 to 102) were referred for suspected myocarditis (n = 5), pericarditis (n = 4), ischemic cardiomyopathy (CMP, n = 27), dilated CMP (n = 4), hypertrophic CMP (n = 3), inflammatory CMP (n = 2), arrhythmia (n = 3), or other diseases (n = 13). A single cMRF slice was acquired in the same basal short-axis view as a 5(3)3-MOLLI T_1_ map and a T_2_-prepared bSSFP T_2_ map whenever either or both were deemed appropriate by the attending physician, and the clinical protocol allowed for the extra scan time. Similarly, cMRF and 4(1)3(1)2-MOLLI were acquired at the same location 20–25 min after the IV injection of 0.2 mmol/kg body weight GBCA (Gadovist, Bayer Healthcare, Leverkusen, Germany). Post-contrast cMRF T_2_ values were obtained but not reported due to low relevance and lack of comparison. The cMRF protocol with shorter acquisition window was kept the same as used for both healthy volunteers and the phantom; the two MOLLI maps were acquired with FOV = 306 × 360 mm^2^, pixel size = 2.0 × 1.4 mm^2^ interpolated to 1.4 × 1.4 mm^2^, and slice thickness = 8 mm, while T_2_prep-bSSFP had FOV = 288 × 360 mm^2^, pixel size = 2.5 × 1.9 mm^2^ interpolated to 1.9 × 1.9 mm^2^, and slice thickness = 8 mm.

In the patients where the native and post-contrast acquisitions were successful with both cMRF and routine techniques, the synthetic extracellular volume (ECV) was also computed:

1 $$\begin{array}{c}ECV=\left(1-\mathrm{Hct}\right) \cdot \frac{\left[\Delta {R}_{1}myocardium\right]}{\left[\Delta {R}_{1bloodpool}\right]},\end{array}$$

where $\Delta {R}_{1}={R}_{1}^{post{\text{-}}contrast}-{R}_{1}^{pre{\text{-}}contrast}$, and ${R}_{1}=1/{T}_{1}$. The value of the hematocrit (Hct) was estimated according to the empirical relationship found by Treibel et al. for MOLLI^35^:

2 $$\begin{array}{c}Hc{t}_{MOLLI}=\left(866.0 \cdot \frac{1}{{T}_{1bloodpool}}\right)-0.1232.\end{array}$$

The heartrate of each patient was recorded for linear regression with the pre- and post-GBCA relaxation times.

### Statistical analysis

All statistical analyses were performed with GraphPad Prism 8 (GraphPad Software, La Jolla, CA, USA; http://www.graphpad.com/scientific-software/prism/) and MATLAB. In vitro, we first computed the mean values and the standard deviation for each considered vial in the phantom. As a measure of accuracy, a Bland–Altman analysis and linear regression against reference values were performed for each technique, with a selected representative heart-rate of 60 bpm. In order to assess the influence of variable heart rates, we computed the coefficient of variation (CoV, standard deviation divided by the mean value) between scans acquired at different synthetic R-R intervals. Multiple linear regressions (one per heart-rate) were also computed for each technique, and Analysis of Covariance (ANCOVA) was used to test whether the slopes differed significantly.

In vivo, precision was defined per subject and segment and the coefficient of variation was again used as a measure of precision. When comparing the average measurements across multiple subjects, the standard deviations of the mean values were reported as absolute values (i.e. in milliseconds). Five datasets from healthy volunteers were segmented by a second observer, in order to assess the inter-observer variation that might be caused by differences in the segmentation. The resulting whole-heart distributions were compared by paired Student’s t-tests. Paired Student’s t-tests were also used to measure the agreement between cMRF and routine techniques. In healthy volunteers, since three slices from the same subject do not represent statistically independent variables, and three techniques were compared, a Bonferroni correction for multiple comparisons was used. When comparing global averages across the whole volunteer population, the three different slices were always averaged first, in order to generate one per-subject point. In all t-tests, P < 0.05 indicated statistical significance. Box and whiskers plots were reported to provide a graphical representation of the results from the multiple t-tests across the myocardial segments.

## Supplementary Information

Supplementary Information.

## Data Availability

The datasets used and analyzed during the current study available from the corresponding author on reasonable request.
