# Supplementary material for: Cardiac MR fingerprinting with a short acquisition window in consecutive patients referred for clinical CMR and healthy volunteers
Source: Sci Rep. 2022 Nov 4;12:18705. doi: 10.1038/s41598-022-23573-3 (PMC9636181; doi:10.1038/s41598-022-23573-3)
Supplement: Supplementary file 1 — Supplementary Information. [file 41598_2022_23573_MOESM1_ESM.docx]

# Supplementary Material

Article title: Cardiac MR Fingerprinting with a short acquisition window in consecutive patients referred for clinical CMR and healthy volunteers

Authors: Simone Rumac, Anna Giulia Pavon, Jesse I. Hamilton, David Rodrigues, Nicole Seiberlich, Juerg Schwitter, Ruud B. van Heeswijk

**Supplementary Figure 1.** **Linear regression plots of the mapping techniques in the ISMRM-NIST phantom.** All the fitted equations are reported on the graphs. **A)** The four T_1_ mapping techniques showed good correlation to the reference across a broad T_1_ range and virtually no difference was observed between the two cMRF variants. **B)** Both cMRF sequences match the SE T_2_ relaxation times ranging from 30 ms to 300 ms. T_2_-prepared bSSFP shows a greater underestimation in the higher T_2_ values, most likely due to the non-physiological T_1_ values in the phantom. **C, D)** Comparison with 5(3)3-MOLLI and 4(1)3(1)2-MOLLI, in their respective ranges of interest. In both considered ranges, cMRF confirms the overestimation of T_1_ relaxation times.

**Supplementary Figure 2. The phantom heart-rate variability study. A, C)** The average T_1_ and T_2_ values measured in the NIST phantom across 8 simulated heartrates (R-R intervals ranging from 600ms to 1300ms). The rightmost column is the gold-standard relaxation time and whiskers represent the standard deviation in milliseconds. **B, D)** The corresponding coefficients of variation expressed as a percentage. Both variations of cMRF showed CoV lower than 2% and 8% in each T_1_ and T_2_ vial, respectively.

**Supplementary Figure 3. Linear regression plots at different heartrates.** The legend indicates different R-R intervals in milliseconds. The P values obtained from the ANCOVA tests are reported in their respective plot. **A-B)** Linear regressions for T_1_ measured with cMRF_154ms_ and cMRF_250ms_, respectively. **C-D)** T_2_ linear regressions for cMRF_154ms_ and cMRF_250ms._ **E-F-G)** The linear regressions for the corresponding routine techniques.


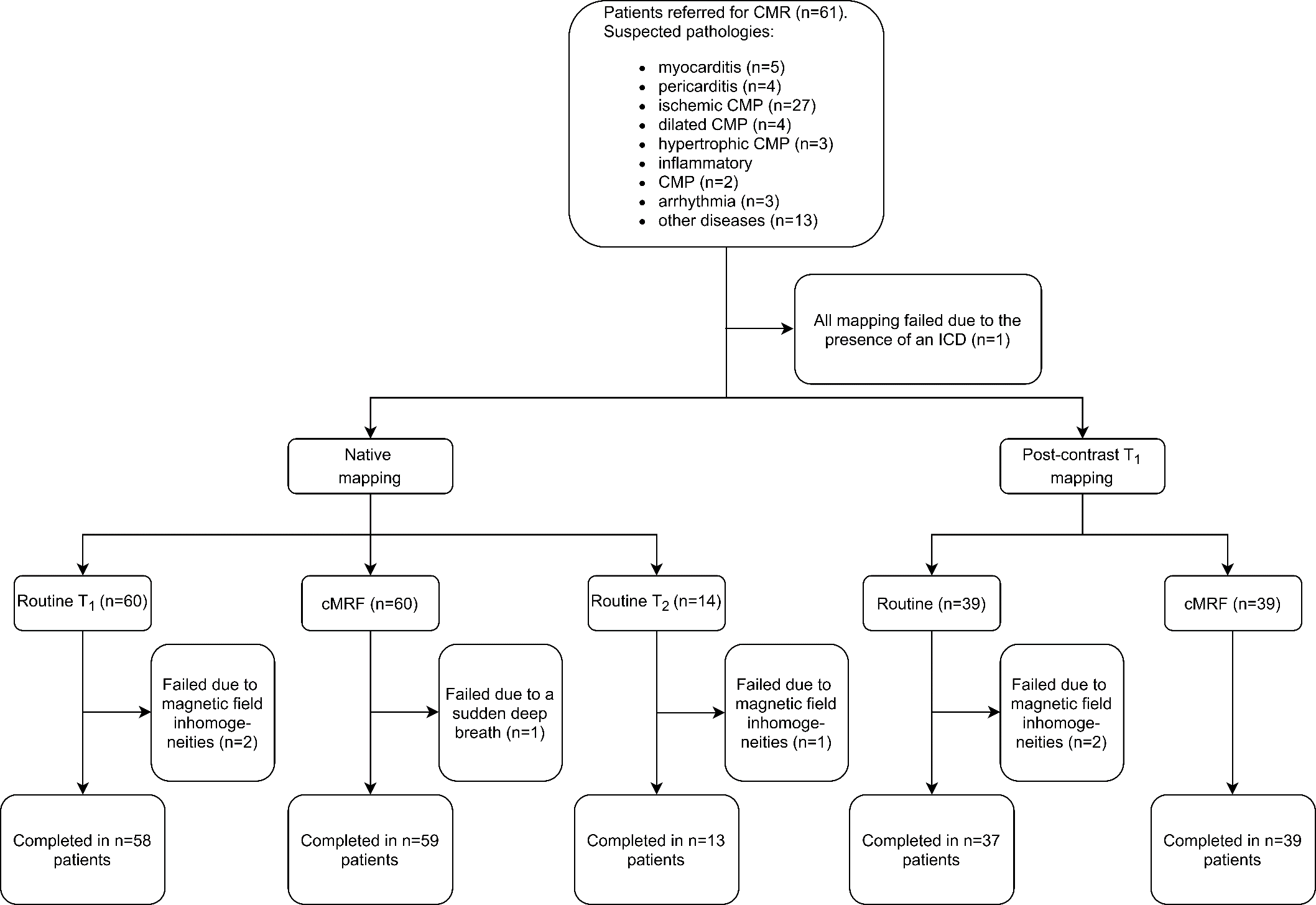


**Supplementary Figure 4.** Flow chart of the patient recruitment, including dropouts, application of the mapping sequences, and completion of the maps.

**
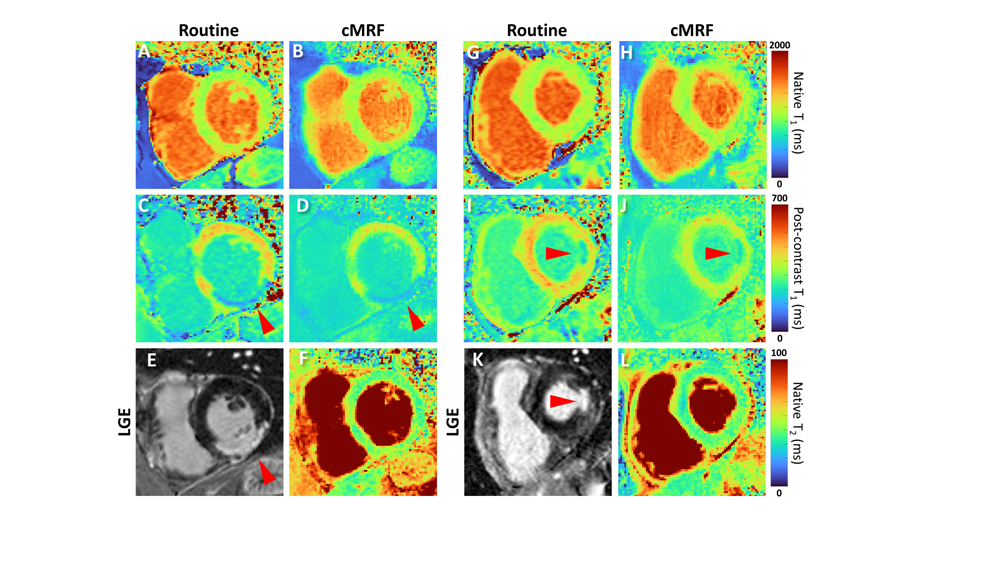
**

**Supplementary Figure 5. cMRF and routine myocardial parametric mapping in two patients. A-F)** 66 year-old male patient with inferior transmural myocardial scar (red arrowhead) after infarction, which is confirmed in the LGE image (E). **G-L)** 51 year-old male patient with a lateral subendocardial scar (red arrowhead) after infarction. Neither T_2_ map shows significant elevation, indicating the absence of edema in these two patients with old post-ischemic scars.

**Supplementary Figure 6. Linear regressions of the patient heartrates with the relaxation times (A-C) and their standard deviations (D-F).** None of the regressions was statistically significant. It should be noted that all available relaxation times were used, and not only the ones for which a pair existed in the matched techniques (thus resulting in many cMRF T_2_ values, for example). The significance of each regression is reported in its graph.

| **IR-SE T_1_** | **cMRF - 154ms** | | **cMRF - 250ms** | | | | **5(3)3-MOLLI** | | **4(1)3(1)2-MOLLI** | |
| --- | --- | --- | --- | --- | --- | --- | --- | --- | --- | --- |
|  | **Mean** | **St. Dev.** | **Mean** | | | **St. Dev.** | **Mean** | **St. Dev.** | **Mean** | **St. Dev.** |
| 46.8 | 86.8 | 4.7 | 82.5 | | | 4.4 | 40.4 | 12.6 | 60.0 | 40.4 |
| 96.4 | 120.0 | 0.0 | 120.0 | | | 0.0 | 81.6 | 7.9 | 80.7 | 6.2 |
| 147.0 | 160.5 | 2.1 | 159.8 | | | 1.5 | 130.5 | 8.6 | 126.1 | 8.3 |
| 170.6 | 230.7 | 2.6 | 227.9 | | | 4.2 | 202.0 | 10.2 | 200.1 | 11.3 |
| 266.0 | 308.9 | 8.8 | 304.1 | | | 7.2 | 275.9 | 7.9 | 272.6 | 6.0 |
| 404.3 | 450.0 | 14.9 | 450.0 | | | 14.5 | 420.6 | 17.1 | 411.4 | 13.5 |
| 547.3 | 596.1 | 19.9 | 610.0 | | | 23.2 | 576.4 | 26.3 | 542.1 | 58.4 |
| 724.5 | 807.6 | 40.7 | 813.0 | | | 41.8 | 753.6 | 32.6 | 712.8 | 72.4 |
| 949.4 | 1065.6 | 52.2 | 1075.6 | | | 60.5 | 993.7 | 23.2 | 961.1 | 22.9 |
| 1221.1 | 1382.7 | 40.4 | 1395.9 | | | 37.3 | 1263.0 | 56.9 | 1198.7 | 41.5 |
| 1501.5 | 1644.3 | 25.6 | 1633.4 | | | 26.4 | 1529.8 | 27.9 | 1436.7 | 11.3 |
| 1810.8 | 1948.2 | 34.3 | 1958.9 | | | 41.5 | 1904.8 | 30.5 | 1708.2 | 22.6 |
| 2084.8 | 2283.6 | 38.1 | 2284.4 | | | 47.2 | 2244.2 | 56.1 | 1959.4 | 15.6 |
| 2238.5 | 2540.8 | 26.5 | 2587.8 | | | 37.5 | 2535.8 | 29.9 | 2215.3 | 29.6 |
| **SE T_2_** | **cMRF - 154ms** | | | **cMRF - 250ms** | | | **T_2_prep-bSSFP** | |  |  |
|  | **Mean** | **St. Dev.** | | **Mean** | **St. Dev.** | | **Mean** | **St. Dev.** |  |  |
| 7.1 | 7.3 | 2.6 | | 9.7 | 2.9 | | 135.4 | 14.1 |  |  |
| 9.8 | 11.5 | 2.3 | | 13.4 | 3.3 | | 87.6 | 8.1 |  |  |
| 13.9 | 16.0 | 2.1 | | 18.2 | 2.7 | | 64.2 | 9.2 |  |  |
| 21.6 | 23.9 | 1.3 | | 25.4 | 2.1 | | 51.6 | 4.6 |  |  |
| 28.8 | 32.0 | 1.8 | | 35.2 | 2.6 | | 52.4 | 5.5 |  |  |
| 46.1 | 47.6 | 1.7 | | 50.2 | 2.3 | | 59.3 | 2.9 |  |  |
| 66.3 | 64.6 | 1.6 | | 67.9 | 2.5 | | 77.0 | 5.6 |  |  |
| 93.4 | 90.7 | 2.7 | | 94.1 | 4.3 | | 100.3 | 7.8 |  |  |
| 133.0 | 129.8 | 5.9 | | 135.9 | 7.5 | | 137.9 | 11.2 |  |  |
| 186.5 | 193.4 | 7.1 | | 201.8 | 8.7 | | 180.0 | 18.5 |  |  |
| 251.9 | 263.0 | 10.7 | | 272.3 | 9.1 | | 224.7 | 12.1 |  |  |
| 362.8 | 358.6 | 18.5 | | 380.0 | 12.9 | | 286.3 | 17.7 |  |  |
| 492.1 | 494.4 | 11.5 | | 499.2 | 4.0 | | 348.6 | 26.6 |  |  |
| 656.6 | 500.0 | 0.0 | | 500.0 | 0.0 | | 403.9 | 13.5 |  |  |

**Supplementary Table 1.** **Phantom comparison of the applied mapping techniques to the reference.** The ISMRM-NIST phantom (QalibreMD, Boulder, CO, USA. System Standard Model 130) is a 200 mm spherical phantom comprised of the following elements: fiducial (4 spheres), T_1_, T_2_ and proton density arrays (14 spheres each). T_1_ arrays were created by doping deionized water with NiCl_2_, while the T_2_ array vials are filled with MnCl_2_ doped deionized water. The reference measurements for each vial are reported, together with the mean and standard deviation as measured by each technique in each vial. The lack of variation in the highest-T_2_ vial is caused by the upper limit of T_2_=500ms in the dictionary.

**Supplementary Paragraph 1. cMRF description**

The cMRF pulse sequence was ECG-triggered with a 255 ms or 154 ms diastolic acquisition window, and data were acquired during a 15-heartbeat breath-hold with a 192 × 192 matrix, 300 mm^2^ field of view (FoV), and 1.6 × 1.6 × 8.0 mm^3^ spatial resolution. A fast imaging with steady-state free-precession (FISP) readout was used with an unbalanced gradient moment along the slice-select axis after each TR. The 15 heartbeat scan is broken into three 5-heartbeat segments, with a scan window in each heartbeat. Each scan window was preceded by an inversion pulse (TI 21 ms) or a T_2_-preparation pulse (echo times of 30, 50, or 80 ms); this series is repeated three times over the 15-heartbeat scan. During the scan window, RF excitations were applied with variable flip angles between 4–25 degrees and a constant relaxation time / echo time (TR/TE) of 5.3/1.4 ms. Sinc-shaped RF pulses were used with a duration of 0.8 ms and time bandwidth product 2. The k-space data were acquired using a variable density spiral trajectory with 29 or 48 interleaves (cMRF_154ms_ and cMRF_250ms_, respectively) that rotated by the golden angle every TR.

The detailed procedure of our compressed MRF with randomized SVD (rSVD-MRF) method can be described as follows. Consider an MRF dictionary$D\in\mathbb{C}^{m \times n}$, with *m* the number of time frames and *n* the number of tissue-type parameters. Let $\Omega\in\mathbb{C}^{n \times k}$be a Gaussian matrix whose entries are drawn from an independent and identically distributed normal distribution with mean 0 and variance 1, where *k* is the low rank desired. We embed the tissue-type dimension $n$into a much lower dimension *k* by $Y={(DD^{*})}^{q}D\Omega$with the power iteration index$=1, 2, \ldots$ , where ${(\cdot)}^{*}$ denotes the conjugate transpose operator, and carry out $QR$ factorization$Y=QR$. Next, form the matrix $B=Q^{*}D$and compute its SVD $B=ǓSV.$Finally, to obtain an approximation of the left singular vectors, compute the matrix $U=QǓ.$We then obtain a rank $k$approximation to the dictionary $D \approx USV^{*}$, where the dimensions of$U$, $S$, and $V$ are$m x k$, $k x k$, and$n x k$, respectively. The choice of the power iteration index $q$depends on the rate of decay of the magnitude of the singular values of the MRF dictionary. When the spectrum of the dictionary matrix is flat, choosing a nonzero $q$ helps reduce the interference of the singular vectors associated with small singular values in calculation, resulting in smaller approximation error. Specifically, the approximation error can be characterized as:

$$E\parallel D-USV^{*}\parallel_{2} \leq\left( 1+4\sqrt{\frac{2\min\left\{ m,n \right\}}{\frac{k}{2}-1}} \right)^{\frac{1}{2q+1}}\sigma_{\left\lfloor\frac{k}{2} \right\rfloor+1} , k>2,$$

where $\left\lfloor\cdot\right\rfloor$denotes the floor operator. The larger the value of q is, the smaller is the approximation error. Now for some test signal **x**, the pattern matching can be calculated via$maxD^{*}\boldsymbol{x}\approx max(VS^{*})(U^{*}\boldsymbol{x})$. The corresponding tissue property values can then be obtained by examining the index of the maximum against the tissue property value table used for the simulation of the MRF dictionary. Note that in real implementation, one does not need to pre-compute and store the entire dictionary D. Only one tissue property entry at a time is calculated and processed on-the-fly to update Y and B for the calculation of U, S, and V, whose sizes are much smaller than that of the dictionary D, resulting in a significant reduction in the computer memory required.

**Supplementary Paragraph 2. Inter-scan variability**

In one healthy volunteer (Female, 25 year-old) we compared the relaxation times measured in two separate scanning sessions. Three short-axis views were acquired with both cMRF variations (apex, middle, and base of the left ventricle), and the average relaxation times were compared by means of a paired Student’s t-test. The comparison for cMRF_154ms_ produced P=0.75 and P>0.99 for T_1_ and T_2_, respectively. cMRF_250ms_ resulted in P>0.99 and P=0.5, for T_1_ and T_2_, respectively. Therefore, no statistical difference was found in-between sessions.
